# Adult Survivorship of the Dengue Mosquito *Aedes aegypti* Varies Seasonally in Central Vietnam

**DOI:** 10.1371/journal.pntd.0002669

**Published:** 2014-02-13

**Authors:** Leon E. Hugo, Jason A. L. Jeffery, Brendan J. Trewin, Leesa F. Wockner, Nguyen Thi Yen, Nguyen Hoang Le, Le Trung Nghia, Emma Hine, Peter A. Ryan, Brian H. Kay

**Affiliations:** 1 QIMR Berghofer Medical Research Institute, Herston, Queensland, Australia; 2 National Institute of Hygiene and Epidemiology, Hanoi, Vietnam; 3 Institute Pasteur, Nha Trang, Vietnam; 4 School of Biological Sciences, University of Queensland, St. Lucia, Queensland, Australia; 5 School of Biological Sciences, Monash University, Melbourne, Victoria, Australia; University of Perugia, Italy

## Abstract

The survival characteristics of the mosquito *Aedes aegypti* affect transmission rates of dengue because transmission requires infected mosquitoes to survive long enough for the virus to infect the salivary glands. Mosquito survival is assumed to be high in tropical, dengue endemic, countries like Vietnam. However, the survival rates of wild populations of mosquitoes are seldom measured due the difficulty of predicting mosquito age. Hon Mieu Island in central Vietnam is the site of a pilot release of *Ae. aegypti* infected with a strain of *Wolbachia pipientis* bacteria (*w*MelPop) that induces virus interference and mosquito life-shortening. We used the most accurate mosquito age grading approach, transcriptional profiling, to establish the survival patterns of the mosquito population from the population age structure. Furthermore, estimations were validated on mosquitoes released into a large semi-field environment consisting of an enclosed house, garden and yard to incorporate natural environmental variability. Mosquito survival was highest during the dry/cool (January-April) and dry/hot (May-August) seasons, when 92 and 64% of Hon Mieu mosquitoes had survived to an age that they were able to transmit dengue (12 d), respectively. This was reduced to 29% during the wet/cool season from September to December. The presence of *Ae. aegypti* older than 12 d during each season is likely to facilitate the observed continuity of dengue transmission in the region. We provide season specific *Ae. aegypti* survival models for improved dengue epidemiology and evaluation of mosquito control strategies that aim to reduce mosquito survival to break the dengue transmission cycle.

## Author Summary

Prolonged survival of adult mosquitoes is critical to dengue transmission cycles, particularly because it takes time for the dengue viruses to infect the mosquito midgut, replicate and infect the salivary glands before transmission can occur. Reducing mosquito lifespan is one avenue to fight dengue, and may be achievable using mosquito parasites such as a life-shortening strain of *Wolbachia* bacteria. We measured the survival rates of *Aedes aegypti* mosquitoes in a village in central Vietnam during the region's three prevailing seasons. In order to predict the ages of wild-caught mosquitoes, we used a method of predicting mosquito age based on the activity of age-responsive genes. We first tested our estimations on mosquitoes that were released into a large field cage that enclosed a house and yard. The survival patterns of mosquitoes in the village differed between seasons, both in terms of the death rate over time and longevity. We estimate that approximately 92 and 64% of the mosquito population survived long enough to transmit dengue during the dry/cool season (January-April) and dry/hot season (May-August), respectively; however, this was reduced to 29% during the wet/cool season (September-December). The relatively high survival of *Aedes aegypti* year round is likely to be a major contributor to the continuity of dengue transmission in the region and provides rationale for reducing mosquito survival to break the transmission cycle.

## Introduction

How long mosquitoes live has been a question asked by tropical disease researchers for over a century [1]. Mosquito longevity is a limiting factor for the transmission of mosquito-borne pathogens, particularly as the pathogens require a period of time to penetrate the midgut lining from an infectious blood meal, replicate and disseminate through the mosquito and infect the salivary glands to enable transmission. Known as the extrinsic incubation period (EIP), this time period can be long relative to the expected lifespan of mosquitoes. High mosquito survival is therefore critical to transmission cycles [2], [3] and reducing mosquito survival rates is a priority of disease control programs [3]–[5]. There have been several varied estimates of the EIP of the dengue viruses with most variation due to temperature [6]. At 27°C, 8–12 d represented a minimum EIP for dengue strains DEN-2 and DEN-4 in *Ae. aegypti*, however, the majority of transmission occurred 16 d post infection [7]. Unfortunately, the survivorship of wild populations of mosquitoes is seldom estimated, probably because of difficulties associated with predicting the ages of individual mosquitoes. Historically, predicting mosquito age has required delicate dissections to observe changes to ovary morphology [8]–[10]. Unrealistic assumptions are made when translating these markers of reproductive history to the estimation of population survival rates [11]. More recent approaches to mosquito age grading, including gas chromatography of cuticular hydrocarbons [12], [13], transcriptional profiling [14], [15] and near infrared spectroscopy [16], [17], have enabled advances in accuracy and/or throughput.

An island village in central Vietnam has been approved as a pilot release site for *Ae. aegypti* infected with the biological control agent *Wolbachia pipientis*. *Wolbachia* are naturally occurring, maternally transmitted, endosymbiotic bacteria of invertebrates. Recent transinfection of *Ae. aegypti* with *Wolbachia* strains have induced stable phenotypes in the mosquito that have great potential for dengue elimination [18], [19]. These include inhibition of infection of the mosquito by the dengue viruses, cytoplasmic incompatibility (a reproductive manipulation that leads to the spread of *Wolbachia* through uninfected populations) and mosquito life shortening [20]. Field releases of *Ae. aegypti* infected with the *w*Mel strain of *Wolbachia*, which induces high dengue resistance with minimal effects on lifespan, have achieved the establishment of infected populations at three localities surrounding Cairns, Australia [19], [21]. The *w*MelPop strain is more virulent than the *w*Mel strain, causing cell lysis and mosquito life shortening in addition to strong dengue inhibition [18], [22]. Field surveys of the native *Ae. aegypti* population at the central Vietnam pilot site were previously conducted to parameterize invasion models to establish release conditions for *Ae. aegypti* infected with *w*MelPop [23], [24].

We established the age structure of the native *Ae. aegypti* population using the transcriptional profiling method of mosquito age prediction. The method involves measuring the transcription of a selection of known age-responsive mosquito genes using quantitative Reverse Transcriptase – Polymerase Chain Reaction (qRT-PCR). Transcription scores are then input into a multivariate non-parametric model to derive an age prediction. A revised model was recently established using a new combination of four genes [25]. Gene selection was based on the strength of association between transcription and age determined by microarray analysis and robustness of the genes to infection by *Wolbachia* infection and environmental variation. This is the first time this age grading approach has been applied to a wild population of *Ae. aegypti* in a tropical, dengue endemic country. Furthermore, we validated the method using mosquitoes that were maintained in a purpose-built, large, semi-field mosquito habitat which consisted of a mesh cage enclosing an existing house, garden and yard. We provide new insights into the survival characteristics of this mosquito vector with relevance to dengue transmission in the region.

## Methods and Materials

### Human ethics statement

Human ethics approval for allowing colonized (dengue-free) mosquitoes to feed on the investigators within the semi-field habitat was obtained from the QIMR Berghofer Medical Research Institute Human Research Ethics Committee (HREC 1162). Blood feeding was considered to cause a medium risk of allergic reaction and provision was in place that individuals were excluded if they reacted strongly to bites. Written consent was obtained acknowledging the right to refuse or withdraw.

### Study site

Tri Nguyen village is located on Hon Mieu island, Khan Hoa province, central Vietnam (12°11′26′′N, 109°13′31′′E). The island is approximately one km from the mainland coastal city of Nha Trang. It is approximately 1.2 km long (117 ha) and the village stretches for approximately 0.2 km (22 ha) along the western side of the island. Dengue transmission occurs sporadically in the village. There were 22 cases of Dengue Hemorrhagic Fever recorded from 2003 to January 2007 (Ministry of Health, unpublished data). There is no piped water supply on the island, so householders store water in a variety of containers ranging in size from small (<100 L) or standard sized (>100 L) earthenware jars to large molded tanks (>2000 L). Water storage containers are the major larval habitat of *Ae. aegypti* on the island, and these have accounted for 93–100% of the population of pupae [23]. Jeffery and others provide additional description of the village and demographics of the *Ae. aegypti* population [23], [24].

### Semi-field mosquito habitat

**Figure 1:**
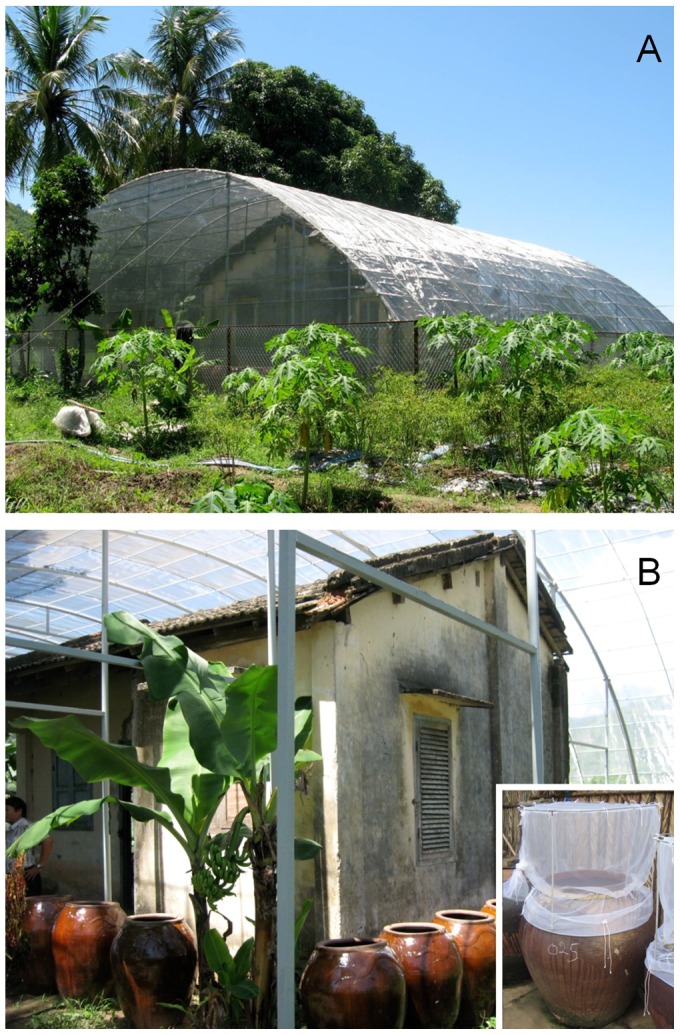
Semi field mosquito habitat for maintaining cohorts of aged *Aedes aegypti*. A. External view of enclosure. B. Internal view of enclosed house and standard sized earthen-ware ceramic water storage jars during conditioning period. Inset. Emergence traps; placed over each jar as soon as pupae were observed. The numbers of females were observed every 24 hours, and when maximum numbers were obtained from a single 24–48 hour period mosquitoes were released.

A semi-field caged habitat was constructed on the outskirts of Nha Trang city to enable experiments on released *Ae. aegypti* derived from locally collected mosquitoes (Fig. 1a). The semi-field facility consisted of a 2-bedroom house (6.3×4.0 m) surrounded by a 0.1 mm mesh stainless steel cage (11.3×12.5×5.0 m) and anteroom, and chain-link security fence around the perimeter (Fig. 1a). Thirty 250 L water storage jars were sourced and placed around the house to facilitate rearing of *Ae. aegypti* immatures under natural conditions (Fig 1b). The jars were purchased either new or used and were conditioned by filling with 150 L of water which was left to stand for approximately 3 wks. The jars were fitted with emergence traps (covered gauze cages above the jars) to prevent the release of emerged adult mosquitoes before the designated release period (Fig 1b inset).

Three experiments were conducted over 8 months covering the three seasons in central Vietnam:

dry/cool (January-April), dry/hot (May-August) and wet/cool (September-December). New colonies of *Ae. aegypti* were established at the National Institute of Hygiene and Epidemiology, Hanoi, from collections of pupae from Tri Nguyen village 6–8 weeks prior to the commencement of each experiment. Colonies were maintained in cages (40×40×40 cm) with access to cotton wool soaked with 10% sucrose and were provided an opportunity to blood feed on the arms/legs of volunteers at 2 d intervals. A container lined with filter paper and approximately 500 ml of water was provided as an oviposition substrate within the cage. Eggs laid on the filter paper were routinely removed, dried and stored. The colonies were increased to a size that would enable >15,000 eggs to be available for flooding on a single day to seed the experimental standard jars. For each experiment, larvae were hatched at the Insitute Pasteur Nha Trang (IPNT) and transferred to the conditioned jars within the semi-field system at densities of approximately 700 instar I larvae per jar. Larvae were fed ground fish food pellets (TetraMin Tropical Tablets, The Rich Mix, Blacksburg, VA, USA) at daily rates of 0.31, 0.71, 1.0 and 1.4 g/jar for I, II, III and IV instars respectively. Food supplementation was performed to overcome density dependence to produce large, synchronous, cohorts of adult mosquitoes emerging within a 48 hour period. Adult mosquitoes were collected using emergence traps and a single cohort of approximately 13,000 mosquitoes were released into the field cage (called semi-field free mosquitoes). 400 mosquitoes were placed inside each of three small cages inside the house (called semi-field caged mosquitoes).

Pupae from the same cohorts were also used to stock three “sentinel cages” situated in Tri Nguyen village (40×40×40 cm; 400 pupae per cage). Mosquitoes in the semi-field habitat and small cages were provided access to a human blood source every second day, from day one. Sucrose feeding stations (pads soaked in 10% sucrose solution) were also provided.

### Tri Nguyen village mosquito collections

#### Sentinel cages

Emerging adult mosquitoes were provided with 10% sucrose and the opportunity to blood feed at two d intervals as described above. Fifteen adult females were collected from the sentinel cages (five mosquitoes per cage) every 4 days, from days 1–29. Mosquitoes were dissected and the head and thorax from each mosquito was placed into 300 µl RNA*later* solution (Ambion, Austin, TX, USA). RNA*later* preserved tissue was stored at 4°C overnight and thereafter at -20°C. One wing from each female was removed and wing length was measured to indicate body size. Wing length was recorded as the distance from the axial notch to the wing tip, excluding the fringe scales [26]. These were subsequently used to calibrate age prediction models for validation experiments and for prediction of the age structure of wild mosquitoes.

#### Wild *Ae. aegypti* collections

Collections of wild adult *Ae. aegypti* were conducted in Tri Nguyen village concurrently with the semi-field habitat experiments. Two hundred households were randomly selected and allocated for mosquito collections using either battery-operated backpack aspirators (BPA) (100 houses) or Biogents sentinel traps (Biogents, Regensburg, Germany; BG traps) (100 houses). BPA collections involved aspirating mosquitoes from typical harborage sites e.g. clothing, under tables, behind furniture etc. over a period of 10 minutes. BG traps were set over a period of 24 hr. Collected female *Ae. aegypti* were dissected and preserved in RNA*later* as described above. Mosquitoes that died during the collection procedure were excluded. The wild specimens were used as samples to estimate the population age structure of *Ae. aegypti* within the Tri Nguyen village. Age structure estimates were produced separately for BPA and BG trap collections and for each season.

### Semi-field mosquito collections

Free flying adult female *Ae. aegypti* were collected from the field cage every two days (12–15 females per day) until no further collections could be made. Mosquitoes were dissected and head and thorax was preserved in RNA*later* as described above. Adult mosquitoes sampled from the semi-field habitat and the semi-field small cages were used as test samples to validate age prediction models.

### Transcriptional profiling

#### cDNA synthesis

The head and thorax from individual stabilized tissue was transferred into a plastic 2 ml screw-cap vial containing 500 µl of TRIsure RNA extraction reagent (Bioline, London, UK). The tissue was homogenized by adding two 3 mm glass beads and shaking the samples for 1.5 min using a Mini-Beadbeater-96 (BioSpec Products, Bartlesville, OK, USA) sample homogenizer. Total RNA was extracted according to the manufacturer's directions for TRIsure, using 100 µl of amylene-stabilized chloroform, 250 µl of isopropyl alcohol and two washes of 1.0 ml 75% ethanol. Total RNA pellets were eluted in 20 µl of RNase-free water. A standard quantity of 350 ng of total RNA per sample was treated to remove genomic DNA contamination using DNase I amplification grade DNase (Invitrogen, Carlsbad, CA) according to the manufacturer's protocol, and reverse transcribed to cDNA using the BioScript reverse transcriptase kit (Bioline). The BioScript protocol was followed with the following modifications: reactions were primed using 0.5 µg custom synthesized anchored oligo-DT_20_ and then incubated with 100 U BioScript and 20 U of RNAse Inhibitor (Bioline) at 45°C for 60 min followed by 70°C for 10 min. The resulting cDNA was diluted 5-fold.

#### Quantitative PCR

Relative quantification was performed of transcripts selected in a recent upgrade to the transcriptional age grading method [25]. These were *AAEL003259* (*Pupal cuticle protein 78E putative*, formerly *Ae-8505*; GenBank accession no. XM_001656550), *AAEL007490* (*conserved hypothetical protein*; XM_001652747), *AAEL008844* (*Calcium-binding protein*, *putative,* previously *Ae-15848*; accession no. XM_001653412), *AAEL014255* (*Aquaporin*, *putative*; XM_001648269) and the reference transcript *AAEL004175* (*40S ribosomal protein S17;* or *RpS17*; XM_001648517). Primer sequences specific to these transcripts have been previously published [25], [27]. Quantitative PCR of individual transcripts was performed in 10 µl reactions including 5 µl of SensiMix *Plus* SYBR (Bioline), 140 ng of cDNA template and 1 µM each of forward and reverse primers. Thermal cycling and transcript quantification was performed using a Rotor-Gene 6000 real time thermocycler (QIAGEN/Corbett, Sydney, NSW, Australia) with a temperature program of 95°C for 10 min, 35 cycles of 95°C for 15 s, 60°C for 10 s and 72°C for 10 s followed by a final ramp from 68°C to 93°C. The fluorescence Take-off value calculated by the Rotor-Gene 6000 software package was taken as the cycle threshold (Ct) value. Duplicate reactions were performed for each transcript and an average Ct value was calculated per mosquito.

### Dengue incidence

Monthly notifications of suspected dengue cases in Nha Trang city were obtained from the National Dengue Program in Central Vietnam. These were divided by the Nha Trang population size for the relevant year to estimate dengue incidence rates.

### Statistical analysis

Relative transcript abundances of *AAEL003259*, *AAEL007490*, *AAEL008844* and *AAEL014255* were determined by calculating log contrast values [15](log_10_ of the ratio of the transcript Ct value to the Ct value of *AAEL004175* [*RpS17*]). Seasonal changes to the expression of the genes was determined by fitting semi-log curves to the relationship between log contrasts and age for each gene and testing the hypothesis that a single curve (or alternatively a season specific curve) best fitted the relationship over different collection periods. Modeling was performed using Graphpad Prism (GraphPad Software, San Diego, CA).

A non-parametric multivariate bootstrapping procedure was used to predict the adult age of female mosquitoes from the four transcript log contrast values. The procedure has previously been described [14], [15], however we have now developed an executable script that can be freely implemented in the R statistical environment. Briefly, the syntax performs canonical redundancy analysis, which extracts a single value for each mosquito (called a redundancy variate) from a combination of log contrast scores that maximizes the correlation of these variables with mosquito age. An age prediction calibration model is constructed by bootstrapping the regression of the redundancy variates of known-age training mosquitoes against their age. A correlation coefficient is calculated that indicates the amount of variation in gene expression measures explained by mosquito age. Y coefficients are calculated indicating the contribution of each gene to the model. A “test” procedure calculates redundancy variates from test (known-age) mosquitoes and predicts age from the calibration model. One thousand age predictions are generated for each individual and the mean of these is reported as the age estimate. Alternatively, a “predict” procedure is provided that predicts the age of unknown-age mosquitoes using the training redundancy variates and generates a frequency histogram of the predicted sample age structure. The R script is freely available from the corresponding author. Season-specific calibration models were constructed from known-age mosquitoes that were maintained within the sentinel cages at Tri Nguyen Village. Adult female *Ae. aegypti* maintained within the semi-field system were used as a test set to determine the accuracy of prediction models. However, mosquitoes could not be collected beyond 17 d old for wet/cool and dry/cool season collections or beyond 11 d old for dry/hot season collections. Therefore, mosquitoes from the semi-field small cages that were collected to 29 d old during each season were used to validate predictions for older mosquitoes. Optimized models were then used to predict the age of wild adult females sampled using the BG traps or by backpack collections from Tri Nguyen.

#### Fitting mortality models to population data

For each season and collection method, the Tri Nguyen village wild-caught mosquitoes were divided into predicted age groups of 0–5 d, >5–10 d, >10 to 15 d etc, to >55 d. To estimate the population at time t, the counts in each age bin, along with the midpoint of each bin was modeled using three mortality models: the exponential model, the Gompertz model, and the logistic model. These three models assume the common form: 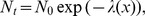



Where 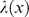 is the hazard function, 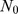 is the population at time 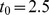, and 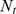 is the population at time t. The hazard functions can be seen in Table 1. Both the Gompertz and Logistic model allow there to be an age dependent mortality rate, while the exponential model assumes constant mortality across time.

**Table 1:** List of survival models tested along with their hazard function.

Model	Hazard function, 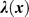
Exponential	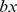
Gompertz	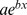
Logistic	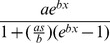

The model parameters were estimated using a non-linear least squares method provided in the nls package for R. As the three proposed models are semi-hierarchical (as noted in [28]), likelihood ratio tests were performed comparing the fit of the exponential to the Gompertz, and if required, the Gompertz to the logistic. Due to the relatively small sample size and number of age categories, a p-value <0.01 was required in the likelihood ratio tests in order to assume the more complex model. The stricter requirement was implemented in order to minimize the chance of over parameterizing the population model.

## Results

### Macro- and micro- climatic conditions

**Figure 2:**
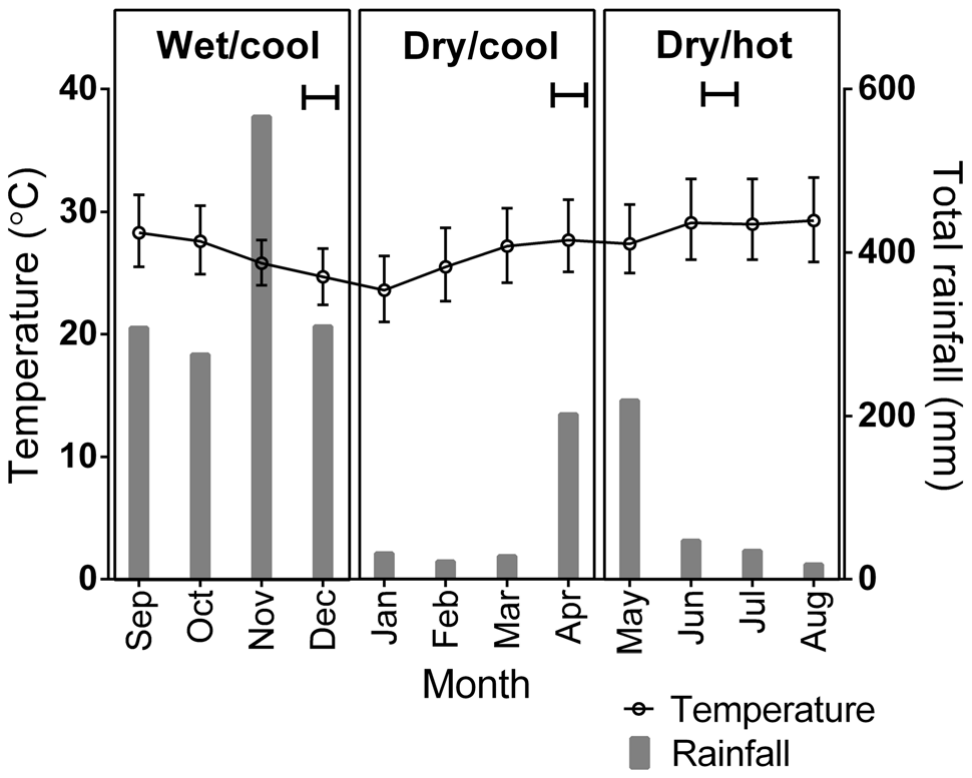
Macro-climatic conditions in Nha Trang, central Vietnam, during the three seasonal field experiments. Brackets indicate the period of semi-field and Tri Nguyen mosquito collections.

Macro-climatic conditions during the three collection periods were consistent with seasonal norms for central Vietnam (Fig. 2). Average ambient temperatures (minimum-maximum) during the experimental collection periods were 24.7°C (22.4–27.0°C) for the wet/cool season, 27.7°C (25.1–31.0°C) for the dry/cool (January-April) season and 28.9°C (24.4–35.0°C) for the dry/hot season (May-August; Fig. S1). Most rainfall occurred during the end of the wet/cool season and start of the dry/cool season collections, reaching a maximum of 71.2 mm per day, and rainfall was largely absent during the dry/hot season collections. Microclimate measurements using data loggers showed that daily temperature profiles indoors within the semi-field house (Fig. S2A) and residences in Tri Nguyen Village (Fig. S2C) were attenuated at the extreme daily minimums and maximums compared to ambient temperature profiles immediately outside these houses (Figs. S2B and S2D, respectively). However, there was close agreement for indoors and outdoors recordings between the semi-field and Tri Nguyen village environments. The same trend was observed for relative humidity measurements (Fig S2E–S2H). The temperature and humidity profiles of microclimates within the semi-field environment were therefore representative of microclimates within the study site at Tri Nguyen Village; they were unaffected by the screened enclosure.

### Mosquito collections

Collections of wild *Ae. aegypti* from Tri Nguyen village using BG traps collected 95–215 female *Ae. aegypti* from 53–109 positive houses per season (Table 2). Collections using backpack aspirators yielded 151–285 females from 74–137 positive houses. The percentages of mosquitoes surviving collection were significantly higher for BPA collections than for BG trap collections over the three seasons (Paired samples t-test; *t* = −5.992, df = 2, *P* = 0.027).

**Table 2:** Collections of wild *Aedes aegypti* samples from Tri Nguyen village over three seasons using BG trap and backpack aspirator (BPA) collection methods.

		Season		
Collection method	Parameter	Wet/cool	Dry/cool	Dry/hot
BG trap	No. houses positive	53	109	102
	Total no. female *Ae. aegypti* collected	95	215	181
	% mosquitoes surviving collection	58.9	83.3	91.2
	Max. no. females analyzed per house	3	3	3
	Total no. samples analyzed for age	46	131	135
BPA	No. houses positive	74	124	137
	Total no. female *Ae. aegypti* collected	151	219	285
	% mosquitoes surviving collection	74.8	98.2	100
	Max. no. females analyzed per house	3	3	3
	Total no. samples analyzed for age	106	194	156
				

Adult mosquitoes released within the semi-field system were collected to a maximum age of 17 d for dry/cool and dry/hot seasons but only to 11 d for the wet/cool season. *Ae. aegypti* females were collected to 29 d old from the semi-field small cages and Tri Nguyen sentinel cages during all seasons.

### Mosquito body size variation

The median wing length of adult females was significantly larger for females maintained during immature stages in standard-jars supplemented with food within the semi-field enclosure than for wild caught females over all seasons (Median test; *P*<0.001; *N* = 1335). The distribution of wing lengths was also more homogenous for jar-reared mosquitoes (Mann-Whitney U; *P*<0.001; *N* = 1335). There was a highly significant effect of season on the mean wing lengths of standard-jar reared females (*F* = 213.345, df = 2, 678, *P*<0.001) with significantly decreasing mean wing lengths from the wet/cool to the dry/hot season (Tukey; P≤0.001; Fig. S3A). Similarly, there was a significant effect of season on the mean wing length of wild caught *Ae. aegypti* females (*F* = 33.317; df = 2, 648; *P*<0.001) with significantly larger wing lengths recorded during the wet/cool than the other two seasons (Tukey; *P*<0.001; Fig. S3B), however the difference between the dry/cool and dry/hot was not significant (Tukey; *P* = 0.161). There was a significant effect attributed to the method used to collect wild females (*F* = 8.250; df = 1, 648; *P* = 0.004); BG traps collected larger females than BPA collections.

### Transcriptional profiling

**Figure 3:**
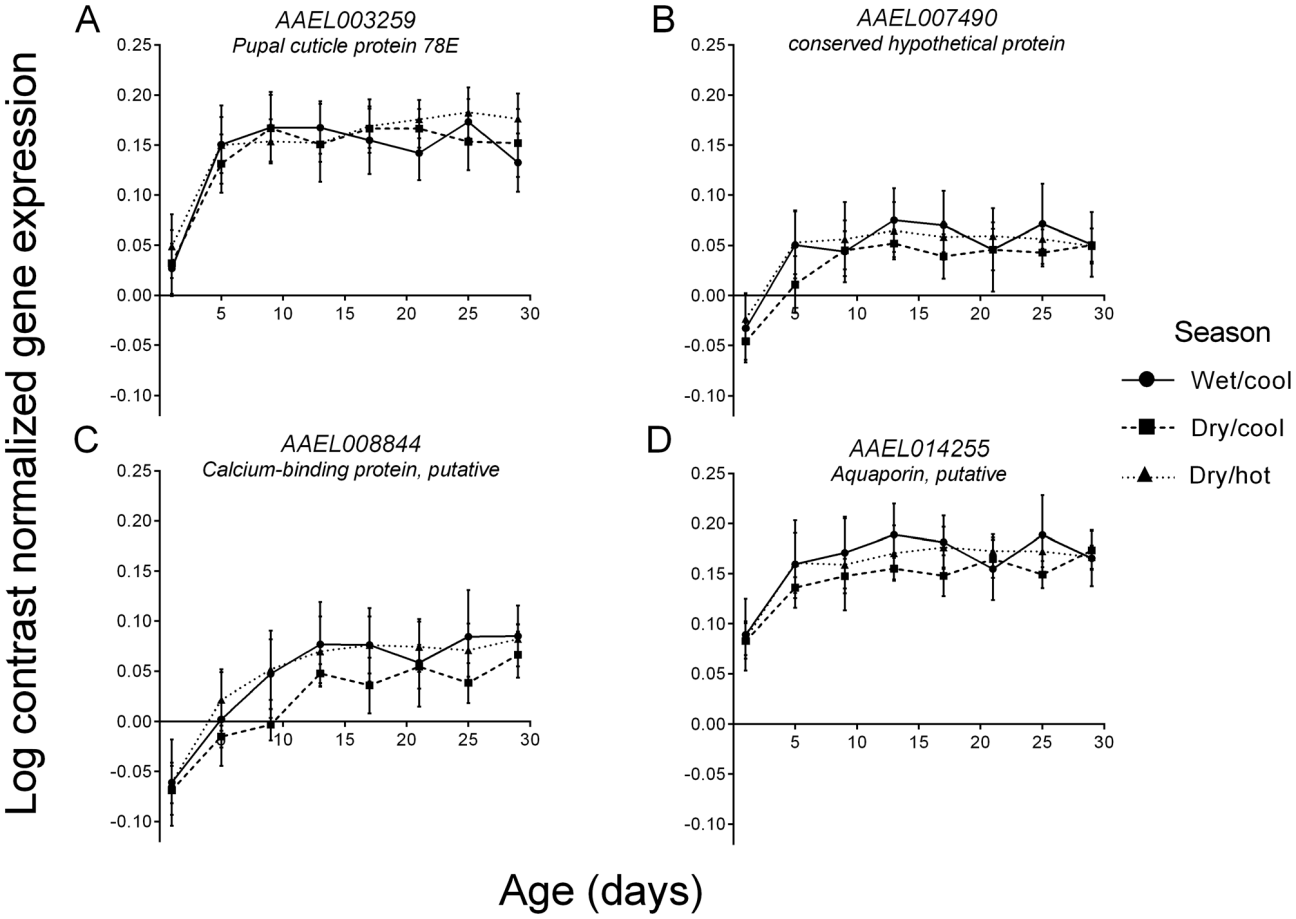
Relative transcription profiles for the four genes used to generate age prediction models from sentinel cage mosquitoes in Tri Nguyen village over three collection periods. A. *AAEL003259* (*Pupal cuticle protein 78E*). B. *AAEL007490* (*conserved hypothetical protein*). C. *AAEL008844* (*Calcium binding protein*, *putative*). D. *AAEL014255* (*Aquaporin*, *putative*). Log contrasts were derived by calculating the log_10_ of the ratio of each gene to the ribosomal protein gene *RpS17*. Upward trends indicate decreasing transcription.

For Tri Nguyen sentinel cage mosquitoes, the strongest relationship between gene transcription and age was observed from gene *AAEL008844* (*Calcium binding protein, putative*. Fig. 3C), which has been the most informative ageing biomarker in previous investigations. Other genes showed curvilinear trends between transcription scores and age. The pattern of *AAEL003259* (*Pupal cuticle protein 78E*) and *AAEL014255* (*Aquaporin*, *putative*) transcription was of an initial rapid increase in the log contrast values at early ages, followed by a stabilization or decrease in log contrast values at older ages (Figs. 3A and 3D). Similar trends were observed for mosquitoes maintained in the semi-field system (Fig. S4). Semi-log curves were fitted to the relationships between log contrast variables and age. For all transcripts, the hypothesis was rejected that a single curve provided the best fit for all seasons, compared to individual curves fitted each season for both the sentinel cage mosquitoes (Fig. S5) and the semi-field free mosquitoes (Fig. S6).

Preliminary age predictions of known age females maintained in small and sentinel cages produced obvious outliers. Further examination of specimens with age prediction residuals > 10 d indicated that these specimens had significantly larger Ct values for the housekeeping gene *RpS17* (*t* = 3.38, df = 84, *P* = 0.001). This was frequently associated with inflated Ct values for all other genes. Abnormally large Ct values for multiple transcripts is most likely due to suboptimal sample quality resulting from factors including RNA degradation from suboptimal storage, transport conditions, or if RNA extraction or cDNA synthesis was inefficient; therefore samples were removed from all datasets if CT values for *RpS17* were > 21 cycles. This produced a loss of 10.7% of the total number of samples (*n* = 1691).

Age prediction models were prepared from gene expression measures from Tri Nguyen sentinel cage females of known ages. Models were first constructed from the log contrast values of all four genes for each season and were assessed based on magnitude of model correlations and y coefficients (Table 3). For all seasons, the ranking of transcripts according to the proportion of contribution that each made to the variance in age was *AAEL008844* (*Calcium binding protein*, *putative*) followed by *AAEL014255* (*Aquaporin, putative*), *AAEL003259* (*Pupal cuticle protein 78E*) and then *AAEL007490* (*conserved hypothetical protein*). Three and two transcript models were also tested by sequentially removing the transcripts with the lowest y coefficients for each season. There was little reduction in model correlations for two or three gene models (Table 3). The highest correlation coefficients for all seasons were always obtained for the four-gene models and so further analyses were limited to four transcript models here.

**Table 3:** Redundancy variate model parameters produced from Tri Nguyen sentinel cage mosquitoes maintained over three seasons.

		Four transcript models			Three transcript models			Two transcript models		
	Transcript	Wet/cool	Dry/cool	Dry/hot	Wet/cool	Dry/cool	Dry/hot	Wet/cool	Dry/cool	Dry/hot
Correlation		0.766	0.861	0.846	0.764	0.861	0.842	0.736	0.861	0.804
Y coefficients	*AAEL003259*	−0.247	−0.055	−0.401	−.238	−0.049	−0.360	×	×	×
	*AAEL007490*	0.134	0.027	0.238	×	×	×	×	×	×
	*AAEL008844*	−1.146	−0.921	−0.980	−1.14	−0.918	−0.988	−1.16	−9.28	−1.151
	*AAEL014255*	1.110	0.218	0.762	1.22	0.236	0.942	1.058	0.175	0.743

×  =  not used in model. Parameters include the y coefficients of the transcripts for four, three and two transcript models indicating the contribution of each gene to the model, and the resulting correlations between each model with mosquito age. *AAEL003259*; *Pupal cuticle protein 78E*, *AAEL007490*; *conserved hypothetical protein*, *AAEL008844*; *Calcium binding protein*, *putative*; *AAEL014255*; *Aquaporin, putative*).

**Figure 4:**
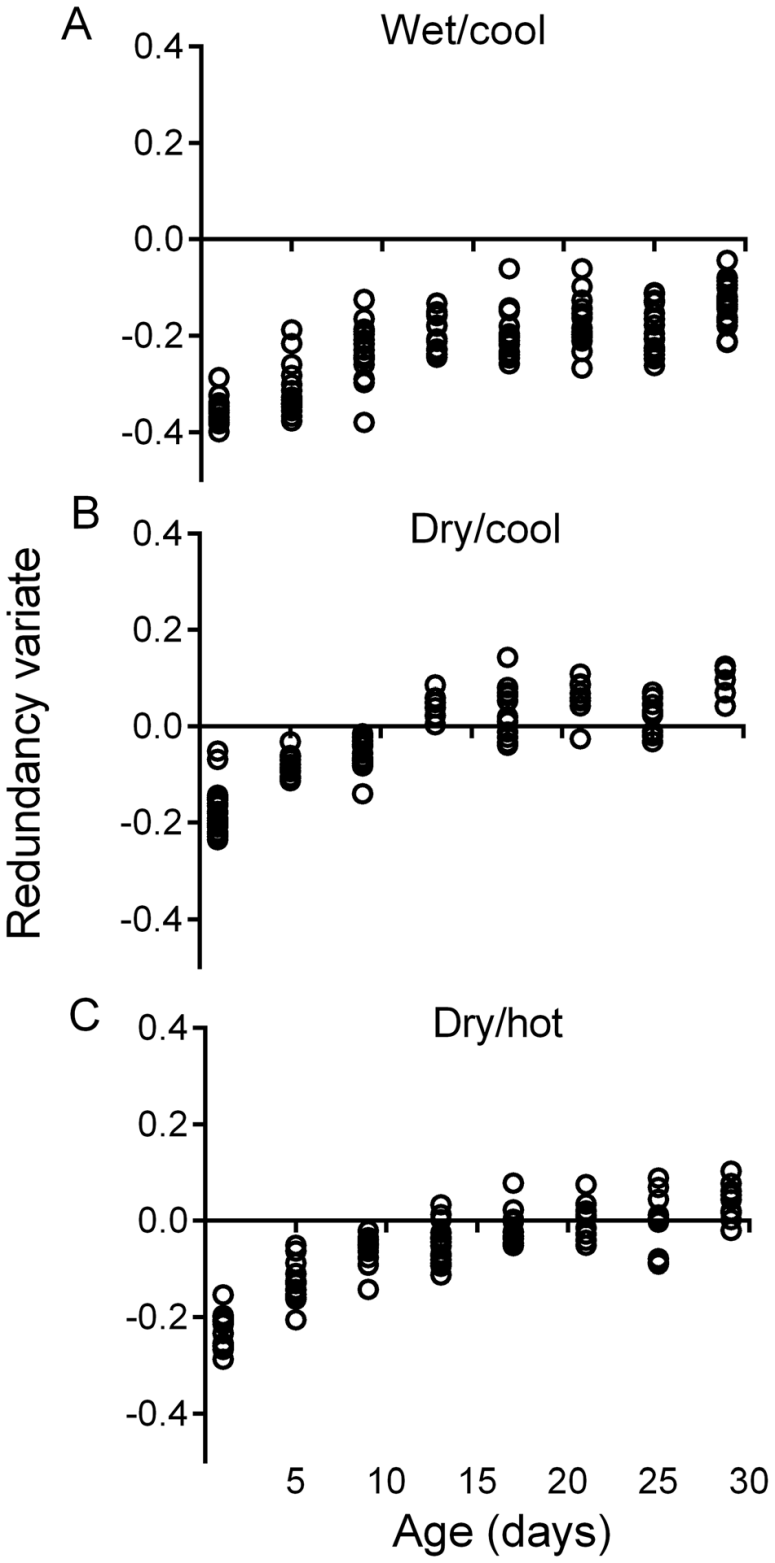
Redundancy variate age prediction models derived from transcriptional scores from Tri Nguyen sentinel cage females. Models were derived from four genes (*AAEL003259*; *Pupal cuticle protein 78E*, *AAEL007490*; *conserved hypothetical protein*, *AAEL008844*; *Calcium binding protein*, *putative*, and *AAEL014255*; *Aquaporin*, *putative*). A. wet/cool season (September-December) model. B. dry/cool season (January-April) model. C. dry/hot season (May-August) model.

Near-linear trends were obtained for the relationships between redundancy variates and mosquito age (Fig. 4) for each season. There was a tendency for the redundancy variates to plateau at ages > 20 d old. The range of redundancy variates during the dry/cool season (January-April; Fig. 4B) and dry/hot (May-August; Fig. 4C) season models were qualitatively similar whereas the wet/cool season (September-December) model was distinct with redundancy variates limited to negative values (Fig. 4A). These models were then applied in test procedures to predict the ages of known age-specimens from semi-field free and semi-field small cage females for each season.

**Figure 5:**
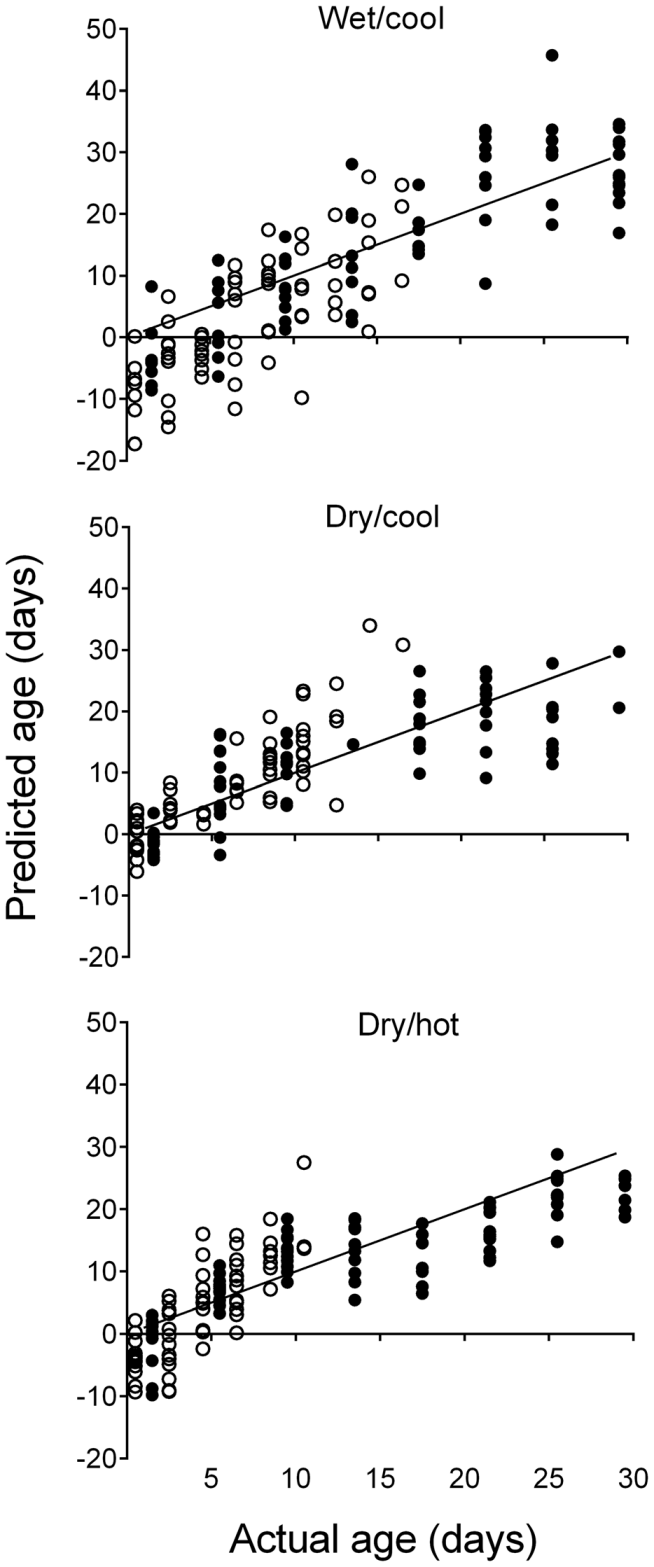
Age predictions of *Aedes aegypti* under seasonal ambient conditions in central Vietnam. The season specific redundancy variate models produced from caged mosquitoes in Tri Nguyen village were applied to estimate the age of free (open circles) and caged (closed circled) semi-field mosquitoes. Points indicate the mean of 1000 bootstrap replications of the redundancy analysis procedure for each sample, taken as the predicted age. The line shows where predicted age equals actual age.

The age prediction capacity of the Tri Nguyen sentinel cage redundancy models was validated on mosquitoes released into the semi-field system and recaptured to maximum ages of 11 to 17 between seasons (Fig. 5; Free). Mean (± SD) residual values for the age predictions were 7.04 (±5.10) d for the wet/cool season, 3.97 (±3.81) d for the dry/cool season, and 4.63 (±3.42) d for the dry/hot season. To validate the performance of the models on older mosquitoes, predictions were then performed on the mosquitoes maintained in small cages in the semi-field facility (Semi-field caged) (Fig. 5; Caged). Wet/cool season predictions for semi-field small cages were unbiased from 1 to 29 d old with mean residuals of 5.83 d (±3.89 d). Dry/cool predictions of caged mosquitoes produced residuals of 4.47 d (±3.25 d). Predictions were most precise during the dry/hot season with residuals of 3.92 d (±3.06 d) from 1 to 29 d old. There was a tendency to overestimate young (≤13 d) mosquitoes and underestimate older (≥17d) mosquitoes.

**Figure 6:**
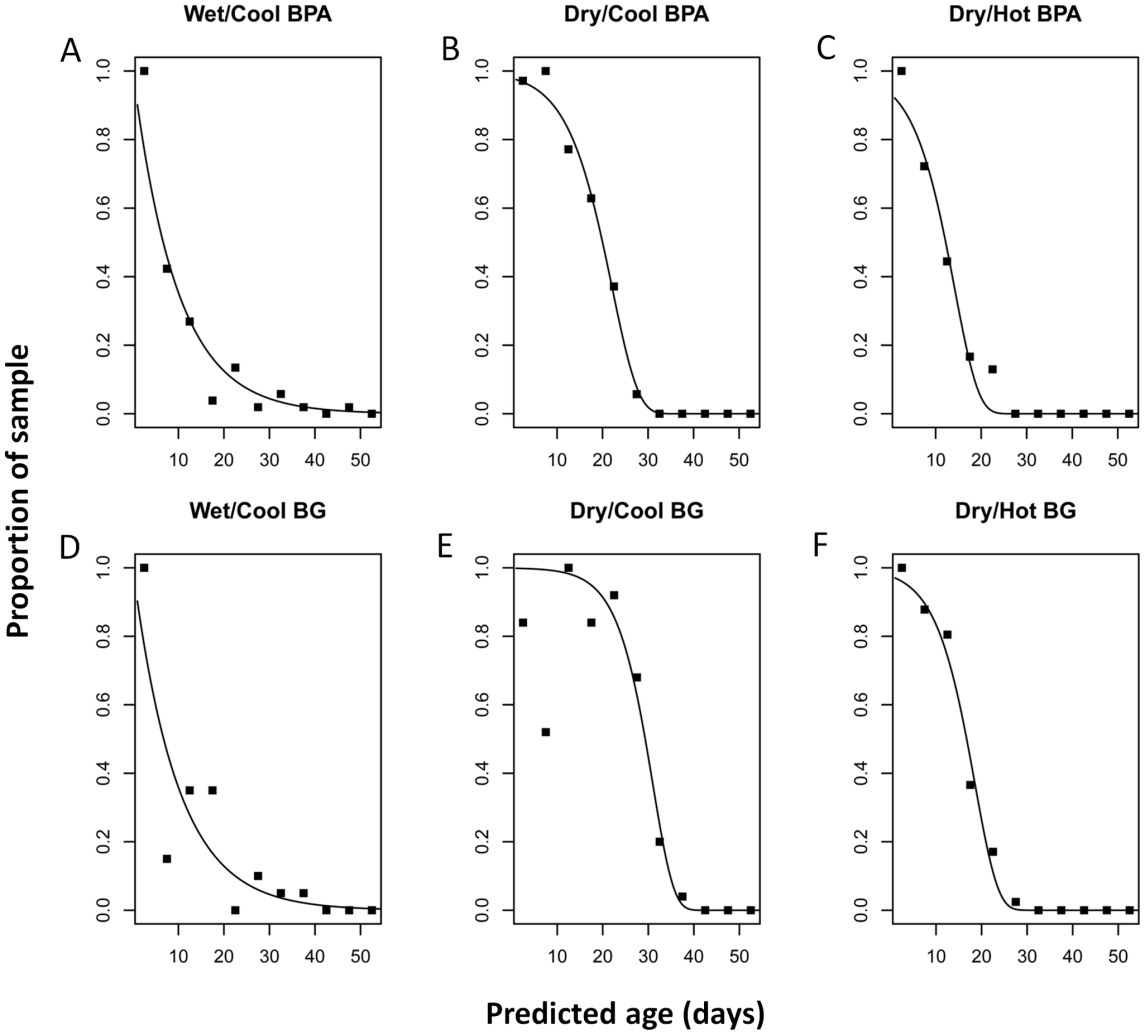
Observed and fitted data for the estimated age distributions of *Aedes aegypti* mosquitoes collected from Tri Nguyen village over three seasons. Age distributions are presented for BG trap (A–C) and backpack aspirator (BPA) collections (D–E). The exponential model was most appropriate for the wet/cool season for both collection methods, while the Gompertz model was most appropriate for dry/cool and dry/hot seasons. Model fit statistics are provided in Table 4.

The ages of wild Tri Nguyen village *Ae. aegypti* collected by both BPA and BG trap methods were predicted using the Tri Nguyen season-specific redundancy variate models. During the wet/cool (September-December) season, the profile of proportions of mosquitoes in different predicted-age classes for BPA and BG trap collections resembled a type III survivorship curve; characterized by a high proportion of 0−5 d old mosquitoes exponentially falling to <5 mosquitoes by 30 d old (Fig. 6A and D). The Gompertz model did not significantly improve the fit of the predicted age group proportions of BPA and BG trap collections during the wet/cool season over an exponential (constant mortality) model (Table 4; Fig. 6 A and D). Constant daily mortality values of 0.102 and 0.104 were estimated for BPA and BG trap collections, respectively. In contrast, the predicted age group proportions for dry/cool season (January-April) and dry/hot season (May-August) collections from both BPA and BG traps formed type I survival curves; characterized by accelerating mortality with age (Fig 6 C, D, E and, F). This was supported by log likelihood tests of the comparison of fitted survival models showing that in all instances, the Gompertz model provided the best fit to the observed age group proportions (Table 4; Fig. 6). Further analysis of the suitability of the Gompertz model compared to another more complex variable mortality model (logistic), was performed. In two instances (dry/cool BG trap and BPA collections; Table 5) the nls algorithm failed to converge to an optimal solution. In these instances the less complex model was chosen. For all other instances, the logistic model did not provide a more appropriate fit over the simpler Gompertz model (Table 5). Season and age-specific hazard rates derived from exponential and Gompertz curves are provided in Fig. S7. Peak mortality occurred at a younger age during the dry/hot season (approximately 20 d) than the dry/cool season (approximately 25−30 d).

**Table 4:** Parameters for the exponential and Gompertz models used to describe the predicted age structures of *Aedes aegypti* collected from Tri Nguyen village.

		Exponential	Gompertz		Likelihood Ratio test
Season	Method	b	a	b	*p*-value
Wet/cool	BPA	0.104	0.106	0.218	0.270
Dry/cool	BPA	0.049	0.021	0.174	<0.01
Dry/hot	BPA	0.078	0.068	0.190	<0.01
Wet/cool	BG	0.102	0.306	0.097	0.042
Dry/cool	BG	0.032	0.001	0.219	<0.01
Dry/hot	BG	0.058	0.025	0.199	<0.01

The likelihood ratio test indicates whether the more complex Gompertz model significantly improved the fit. P-values less than 0.01 indicate the more complex model was more appropriate.

**Table 5:** Parameters for the Gompertz and Logistic models used to describe the predicted age structures of *Aedes aegypti* collected from Tri Nguyen village.

		Gompertz		Logistic			Likelihood Ratio test
Season	Method	a	b	a	b	s	p-value
Wet/cool	BPA	0.106	0.218	0.050	0.374	0.123	0.032
Dry/cool	BPA	0.021	0.174	*convergence not achieved			
Dry/hot	BPA	0.068	0.190	0.049	0.239	0.058	0.016
Wet/cool	BG	0.306	0.097	0.000	1.829	0.794	0.015
Dry/cool	BG	0.001	0.219	*convergence not achieved			
Dry/hot	BG	0.025	0.199	0.013	0.252	0.053	0.032

The likelihood ratio test indicates whether the more complex Logistic model significantly improved the fit. P-values less than 0.01 indicate the more complex model was more appropriate. Convergence not achieved indicates the nls algorithm failed to find an optimal solution.

When the assumptions of a stable age distribution are met (births equal deaths, emigration equals immigration), the age structure functions (N[t]) are equivalent to survival functions (S[t]). By substituting the *a* and *b* parameters into the best fit models for each season, and taking averages between BPA and BG trap collections, 29.1% of the *Ae. aegypti* population had survived until 12 d old (comprising a 2 d pre-feeding period and a 10 d EIP) during the wet/cool season, compared to 91.5% for the dry/cool season and 63.8% for the dry/hot season.

Dengue transmission occurred continuously in the region during the study period as indicated from notifications of suspected dengue cases in neighboring Nha Trang city (Fig. S8). Notifications were highest during the wet/cool season (127.2 suspected cases per 100,000 people; Fig. S8) and dropped to approximately half this level during both the dry/cool (January-April; 63.0 per 100,000) and the dry/hot (May-Aug 2009, 53.9 per 100,000) seasons. Notifications for the dry/hot period unseasonably low when compared with the years immediately before and after the experimental period (Fig. S8).

## Discussion

Prolonged survival of mosquitoes is critical to the transmission cycles of mosquito-borne pathogens [2], [3] but mosquito survival is seldom measured due to difficulties with measuring mosquito age. Here, we provide new insights into the survival of the dengue mosquito *Ae. aegypti* by using transcriptional mosquito age grading for the first time in a tropical dengue-endemic country. We show that in a rural village in central Vietnam, higher survival of *Ae. aegypti* occurs during the dry/cool and the dry/hot seasons occurring during May-October compared to the wet/cool season (September to December). Between 63.9 – 91.5% of the population had survived to an age that they can transmit the dengue virus (12 d) during the dry/cool and dry/hot seasons, whereas this decreased to 29% during the wet/cool season. Our observations that *Ae. aegypti* older than 12 d were present in Tri Nguyen during all three seasons are supported by notifications of suspected dengue cases occurring year round in neighboring Nha Trang city. The survival proportions were not positively correlated with the numbers of suspected dengue cases during the three seasons examined, however notifications were unseasonally low during the dry/hot season. A simple exponential model adequately fitted the estimated population age structure during the wet/cool season, in which survivorship can be summarized by a probability of daily survival of 0.9 (90% of the population surviving from one day to the next). However, during the dry/cool and dry/hot season, Gompertz models, incorporating an age-dependent mortality component, provided significantly better fit to the observed age structures and we provide relevant parameters. This suggests that mortality is driven by exogenous factors such as adverse climatic conditions during the wet/cool season, but that mosquitoes survive long enough to die of senescence related factors during the other seasons.

Our survival trends for *Ae. aegypti* in central Vietnam correlate well with another investigation of *Ae. aegypti* survivorship in a tropical setting where mosquito age was predicted using cuticular hydrocarbon analysis [29]. Gerade et al. found that the survival of *Ae.* aegypti in Thailand was higher during the dry/cool than the rainy season and applied season specific age prediction models for the prediction of mosquito age. In dengue endemic parts of Rio de Janeiro, mark, release and recapture of *Ae. aegypti* indicated that 33–50% of females survived beyond an EIP of 10 days in a low-income, densely populated urban area, whereas only 10% of females survived to transmit dengue in a well-planned, suburban area [30]. Similarly, our results contrast with a recent analysis of the survival of *Ae. aegypti* in Tucson, Arizona, using transcriptional ageing and parity estimation [31]. Only 9% of the population survived for longer than 15 days in this arid environment associated with a high percentage of indoor living.

Our findings of strong associations of *Ae. aegypti* to both age-independent and age-dependent survivorship models for different seasons provide new insights into mosquito survival dynamics. There has been a tendency to assume that the probability of daily survival is equal at all ages in order to simplify survival models. However, assessments of over >100,000 laboratory held *Ae. aegypti* demonstrate that the mosquitoes are subject to senescence, an increasing force of mortality with advancing age [28]. Senescence occurs if the main forces of mortality are age-dependent pathologies such as decreased immune function or detoxification capacity. In the field environment, exogenous factors also contribute to mortality, such as predation and adverse environmental conditions. Such external factors have been hypothesized to shorten lifespan before senescence can take effect [2] [32] or to have an effect in addition to the underlying force of senescence [33]. By releasing and recapturing cohorts of various ages in Thailand, Harrington and others show that recapture rates decreased with age providing evidence that older mosquitoes are also subject to increased mortality in the field [34]. Our analysis based on snapshots of age structure indicate that mosquitoes from a single location can exhibit age dependent and age-independent mortality at different times, suggesting that exogenous factors override the effect of senescence under certain conditions. Further modeling of dry/cool (January-April) and dry/hot (May-August) collections demonstrated that no improvements to the fit of the observed age structures could be achieved by applying a more complex logistic model. This is likely due to small values of mortality deceleration achieved in this study which, due to a semi-hierarchical relationship, renders the two models virtually identical [28].

Calibration and validation of the mosquito age prediction models was performed on mosquitoes exposed to natural environmental fluctuations during each season to improve the predictions for wild mosquitoes. A purpose-built semi-field cage provided an environment in which climatic measurements closely simulated the climate experience by the target population in Tri Nguyen village. Furthermore, mosquito larvae were maintained in standard water storage jars and larvae and captive adults were held under ambient conditions. Both jar-reared and wild caught adults showed similar proportional decreases in body size with increases in ambient temperature. The enclosed environment enabled F2 cohorts of an indigenous strain of *Ae. aegypti* to be released and recaptured at known age points. Mosquito survival within the system was lower than mosquitoes from the same cohort that were maintained in small mosquito cages. Reduced survival in the semi-field system was probably due to predation. Geckos and jumping spiders were abundant inside the house and were actively preying on mosquitoes during the trial, and this could possibly explain the relatively low survivorship of adult mosquitoes inside the semi-field environment (i.e., no individuals recovered older than 11–17 days).

Transcriptional profiling enabled age grading of *Ae. aegypti* mosquitoes under ambient conditions well within the tropical range of the species. We previously determined that temperature significantly influences transcription of *AAEL008844* (*Calcium binding protein*, *putative*) and*AAEL003259* (*Pupal cuticle protein* 78E) genes [35] and accounted for this by producing season-specific redundancy variate age prediction models in the present study. The seasonal changes in climate probably account for the differences between redundancy variate age prediction models. The wet/cool season model was distinct from either the dry/cool and dry/hot models; however the latter were similar to each other. The accuracy of age predictions of test mosquitoes was similar to accuracies achieved for transcriptional age grading of *Ae. aegypti* under semi-field conditions in Cairns, Australia [25]. Mean (SD) differences between predicted and actual ages of ± 3.92 (3.06 d) d to 5.83 (3.89) d between seasons for mosquitoes maintained in small cages in this study compared to ±5.44 (3.84) d for a mixed population of wild type and *Wolbachia* infected *Ae. aegypti* in Cairns. For all seasons, there were only minor improvements to redundancy model characteristics when using four transcripts when compared to model using three, or even two transcripts. This indicates that further time and cost savings may be achieved by streamlining transcriptional analysis to the most informative genes. Gene *AAEL008844* (*Ae. aegypti Calcium binding protein*, *putative*) was always the standout contributor to age prediction models. Four gene redundancy variate models had positive linear associations with age to approximately 13–17 d, after which the relationships leveled out. Improvements in the prediction of older ages will be achieved if models can be constructed that remain linear over the range of ages and seasons investigated, possibly through the incorporation of new transcripts.

There are a number of limitations to the application of age grading to the estimation of population age structure and underlying survival rates. Assumptions of this approach are that age structure of the population is stable over time, recruitment to the population equals loss, and all ages are assumed to be sampled with equal probability. Environmental factors including isolated patches of heavy rainfall could trigger large synchronized *Ae. aegypti* larval hatching events that lead to a pulse in adult numbers successively moving through age classes. Such events could lead to a distortion of the population age structure. A large rainfall total was recorded during the month prior to the wet/cool (September-December) collection period and isolated patches of rain occurred during the wet/cool and dry/cool season. In contrast, the dry/hot period was the most climatically stable with almost no rainfall occurring. Our conclusions are strengthened by observations of similar age structure estimates for each season from independent samples collected by BPA and BG traps, an experimental design used to minimize age collection bias from any one collection method. BG trapping, though convenient, has been associated with a bias against young nulliparous mosquitoes [36]. This may explain a decrease in <5 d old mosquitoes in BG trap collections during the dry/cool season. The body sizes of jar-reared adults, demonstrated from mean wing length, were larger than wild-caught adult mosquitoes that had developed naturally in water storage containers within Tri Nguyen village. This is likely due to the supplementation of food sources for the reared larvae, which was required to produce sufficiently large cohorts (approximately 15,000) of mosquitoes emerging within a short time window to stock the semi-field environment and cages. Food supplementation to the training and test mosquitoes is also likely to have alleviated natural density dependent factors that affect larval survival [37], [38] and potentially transcription [39]. These factors potentially differentiated the reared mosquitoes used to derive the age prediction models from the wild caught samples for which age was predicted. However, we have previously shown that varying the quantity of food supplemented to larvae, does not significantly affect the transcription of two genes used in our study, including the most informative gene (*AAEL008844*; *Calcium binding protein*, *putative* [35]).

This study provides insights into the survivorship of *Aedes aegypti* in a tropical location, which is a critical, but understudied, factor affecting dengue transmission. Mosquito survivorship in a rural village in central Vietnam remained sufficiently high over three seasons to support dengue transmission year round. This provides scope for the application of mosquito life-shortening strategies to break the persistence of dengue in the region. Furthermore, seasonal changes to the pattern of *Ae. aegypti* survivorship, from being age independent to age dependent, should be factored into transmission models to improve dengue epidemiology.

## Supporting Information

Figure S1 Daily meteorological recordings from Nha Trang, central Vietnam, during the experimental periods. Points show mean (error bars  =  min-max) daily temperature recordings. Columns show average rainfall recordings. Data were obtained from http://www.tutiempo.net/en/Climate/Nha_Trang/488770.htm.
(TIF)

Figure S2 Microclimate measurement from within the semi-field system and from Tri Nguyen village. A, C. Temperature recordings within the enclosed house in the semi-field system and within a home in Tri Nguyen village, respectively. B, D. Temperature recordings outside the enclosed house (within the semi-field system) and outside a home within Tri Nguyen village. E, G. Relative humidity recordings within the enclosed semi-field environment house and within a Tri Nguyen Island home. F, H. Relative humidity recordings outside the enclosed semi-field house and outside a Tri Nguyen Island residence.
(TIF)

Figure S3 Seasonal variation in the wing lengths of adult *Ae. aegypti* evaluated during the investigation. A. Wing lengths of Tri Nguyen F1 *Ae. aegypti* maintained as larvae in standard-jars supplemented with food to achieve synchronous adult emergence. Boxes show the median and interquartile range. B. Mean ± SD wing lengths of wild Tri Nguyen *Ae. aegypti* collected as adults using either backpack aspirator collections or BG trap collections. Lines indicate significant differences in mean length between months (Tukey; P≤0.001).
(TIF)

Figure S4 Relative transcription profiles for the four genes used to generate age prediction models measured from semi-field small cage mosquitoes over three collection periods. A. *AAEL003259* (*Pupal cuticle protein 78E*). B. *AAEL007490* (*conserved hypothetical protein*). C. *AAEL008844* (*Calcium binding protein*, *putative*). D. *AAEL014255* (*Aquaporin*, *putative*). Log contrasts were derived by calculating the log_10_ of the ratio of each gene to the ribosomal protein gene *RpS17*. Upward trends indicate decreasing transcription.
(TIF)

Figure S5 Semi-log curves fitted to the transcriptional profiles of the Tri Nguyen sentinel cage mosquitoes over three seasons. A. *AAEL003259* (*Pupal cuticle protein 78E*). B. *AAEL007490* (*conserved hypothetical protein*). C. *AAEL008844* (*Calcium binding protein*, putative). D. *AAEL014255* (*Aquaporin*, putative). *P* values indicate the significance of the difference between the regression statistics for individual semi-log models fitted to data for each season (lines) compared to a single model fitted to all data.
(TIF)

Figure S6 Semi-log curves fitted to the transcriptional profiles of ageing biomarker gene from semi-field small cage mosquitoes over three seasons. A. *AAEL003259* (*Pupal cuticle protein 78E*). B. *AAEL007490* (*conserved hypothetical protein*). C. *AAEL008844* (*Calcium binding protein*, putative). D. *AAEL014255* (*Aquaporin*, putative). *P* values indicate the significance of the difference between the regression statistics for individual semi-log models fitted to data for each season (lines) compared to a single model fitted to all data.
(TIF)

Figure S7 Hazard rates describing the survivorship of *Ae. aegypti* females in Tri Nguyen village during three seasons. A. *AAEL003259* (*Pupal cuticle protein 78E*). B. *AAEL007490* (*conserved hypothetical protein*). C. *AAEL008844* (*Calcium binding protein*, putative). D. *AAEL014255* (*Aquaporin*, putative). Survivorship of wet/cool season (September-December) mosquitoes was best described by an exponential model (hazard is constant), whereas survivorship for dry/cool (January-April) and dry/hot (May-August) mosquitoes was best described by Gompertz models (hazard is variable with age). The hazard rates were calculated using the hazard functions from Table 1.
(TIF)

Figure S8 Notifications of suspected dengue cases in Nha Trang, Central Vietnam. Arrows indicate the timing of experiments. W/C; wet/cool season (September-December), D/C; dry/cool season (January-April), D/H; dry/hot season (May-August).
(TIF)
