# Supplementary figures and images for: Adult Survivorship of the Dengue Mosquito Aedes aegypti Varies Seasonally in Central Vietnam
Source: PLoS Negl Trop Dis. 2014 Feb 13;8(2):e2669. doi: 10.1371/journal.pntd.0002669 (PMC3923839; doi:10.1371/journal.pntd.0002669)

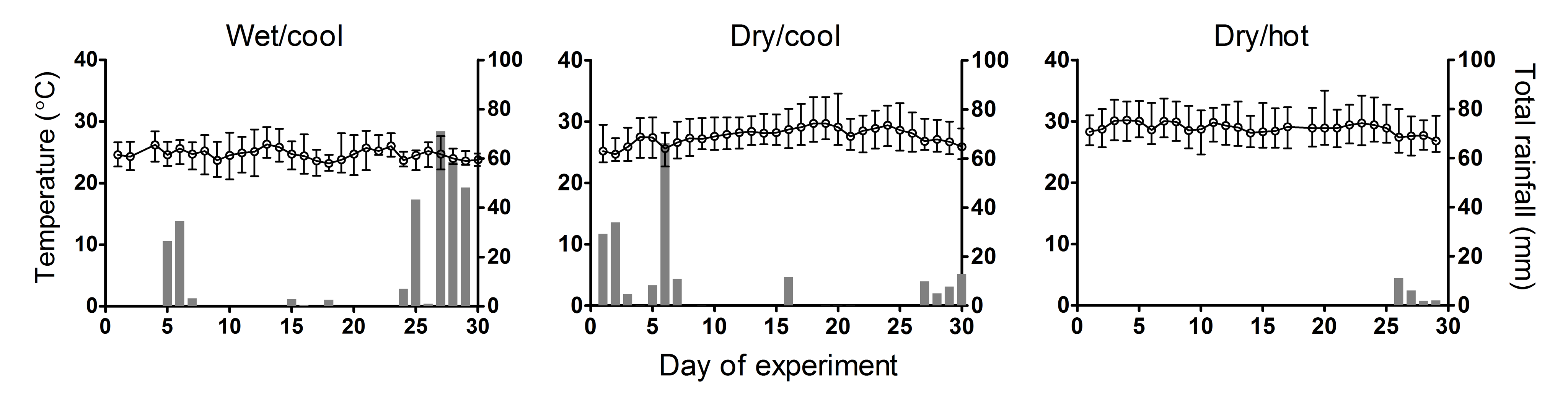

Supplement: Figure S1 — Daily meteorological recordings from Nha Trang, central Vietnam, during the experimental periods. Points show mean (error bars = min-max) daily temperature recordings. Columns show average rainfall recordings. Data were obtained from http://www.tutiempo.net/en/Climate/Nha_Trang/488770.htm. (TIF) [file pntd.0002669.s001.tif]

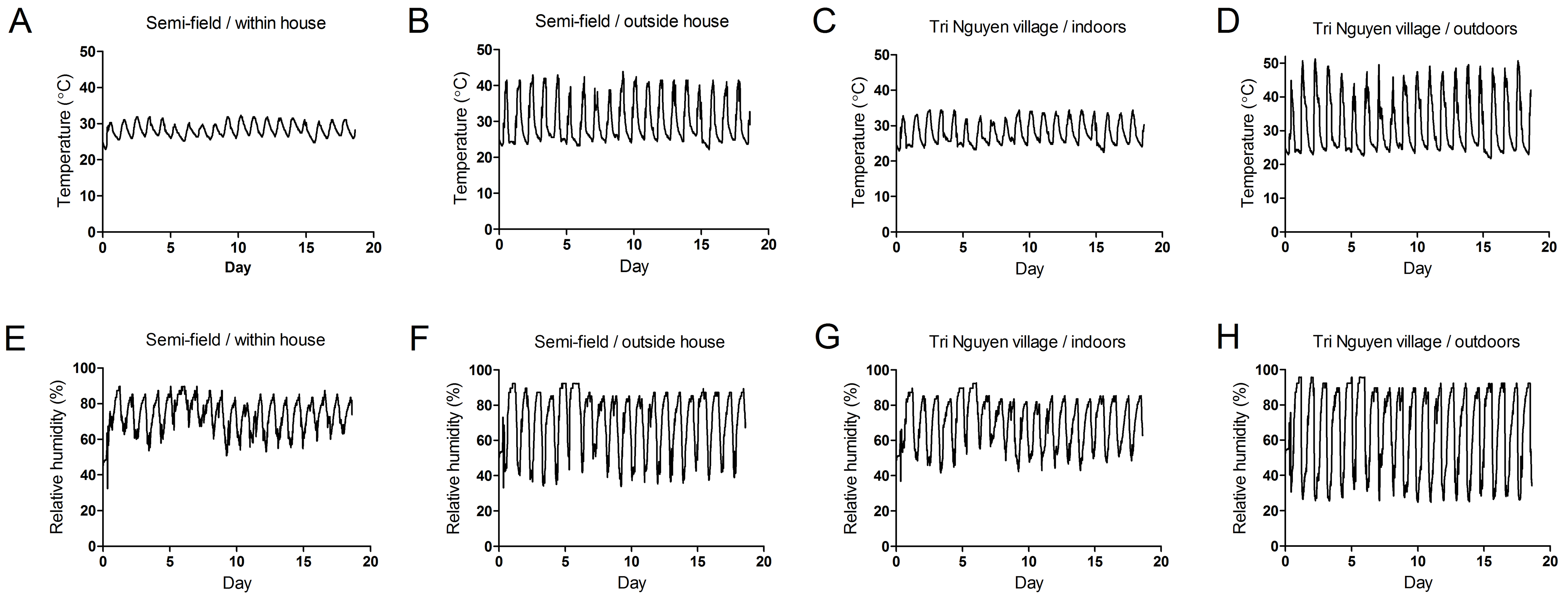

Supplement: Figure S2 — Microclimate measurement from within the semi-field system and from Tri Nguyen village. A, C. Temperature recordings within the enclosed house in the semi-field system and within a home in Tri Nguyen village, respectively. B, D. Temperature recordings outside the enclosed house (within the semi-field system) and outside a home within Tri Nguyen village. E, G. Relative humidity recordings within the enclosed semi-field environment house and within a Tri Nguyen Island home. F, H. Relative humidity recordings outside the enclosed semi-field house and outside a Tri Nguyen Island residence. (TIF) [file pntd.0002669.s002.tif]

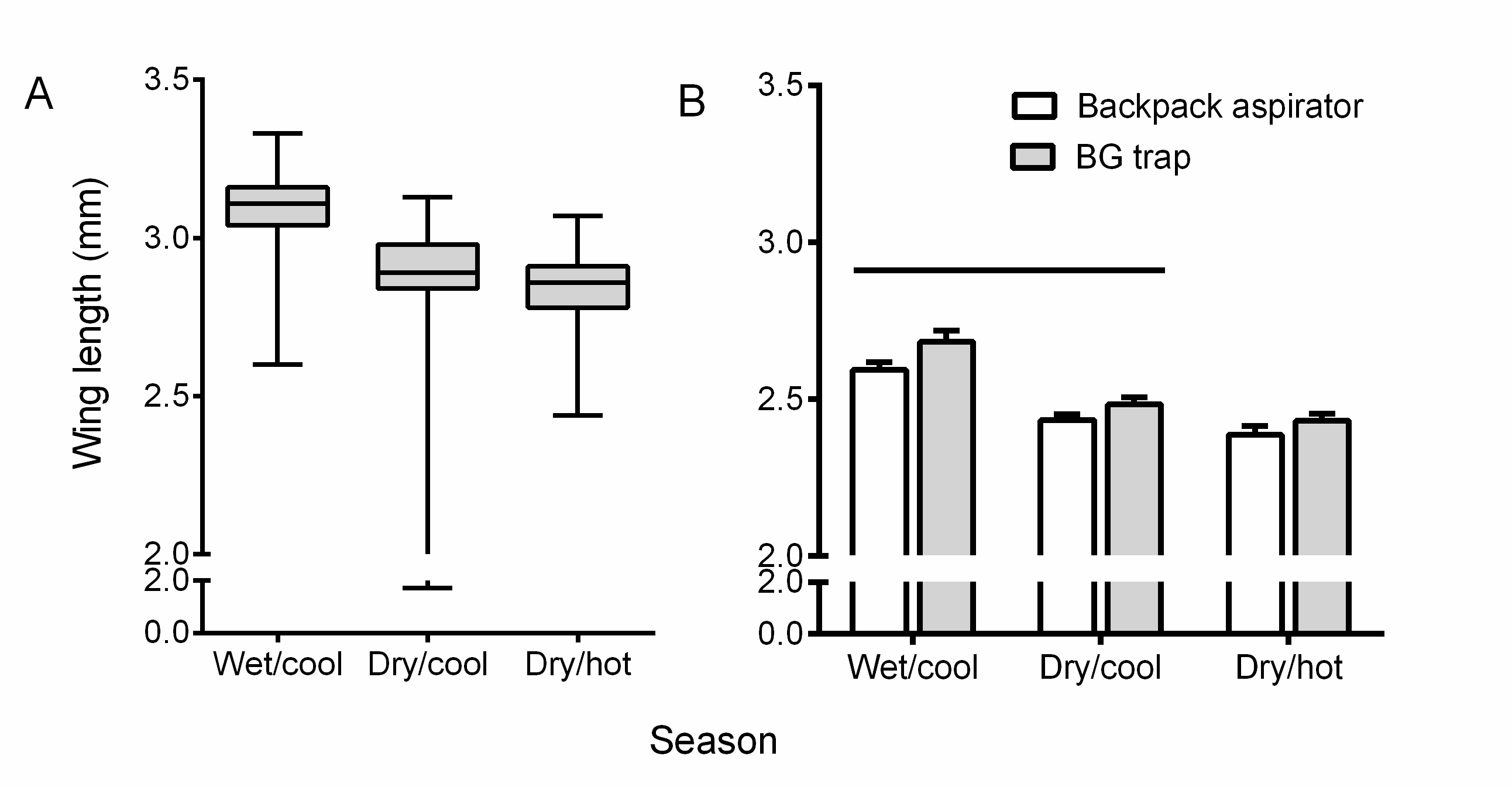

Supplement: Figure S3 — Seasonal variation in the wing lengths of adult Ae. aegypti evaluated during the investigation. A. Wing lengths of Tri Nguyen F1 Ae. aegypti maintained as larvae in standard-jars supplemented with food to achieve synchronous adult emergence. Boxes show the median and interquartile range. B. Mean ± SD wing lengths of wild Tri Nguyen Ae. aegypti collected as adults using either backpack aspirator collections or BG trap collections. Lines indicate significant differences in mean length between months (Tukey; P≤0.001). (TIF) [file pntd.0002669.s003.tif]

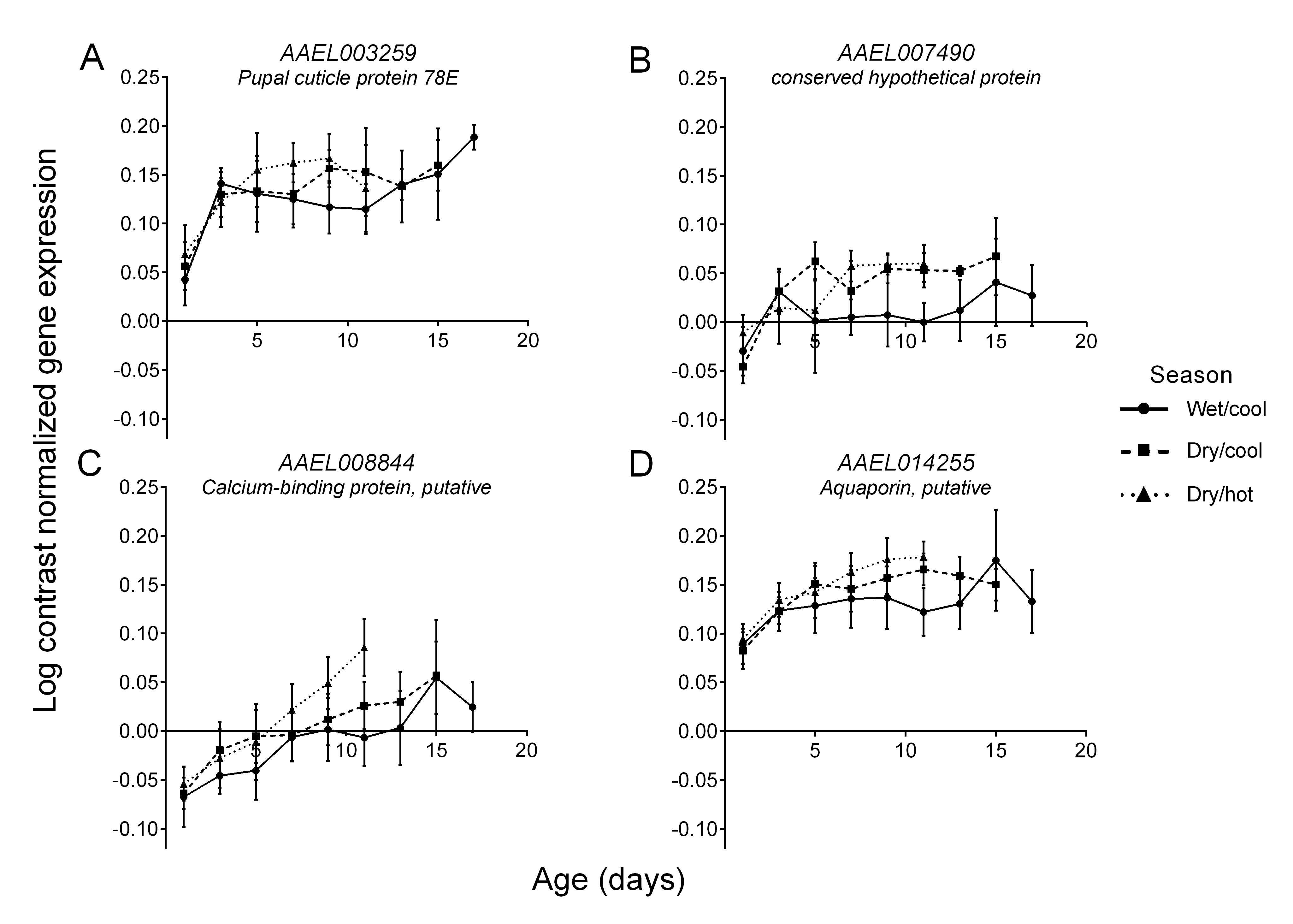

Supplement: Figure S4 — Relative transcription profiles for the four genes used to generate age prediction models measured from semi-field small cage mosquitoes over three collection periods. A. AAEL003259 (Pupal cuticle protein 78E). B. AAEL007490 (conserved hypothetical protein). C. AAEL008844 (Calcium binding protein, putative). D. AAEL014255 (Aquaporin, putative). Log contrasts were derived by calculating the log10 of the ratio of each gene to the ribosomal protein gene RpS17. Upward trends indicate decreasing transcription. (TIF) [file pntd.0002669.s004.tif]

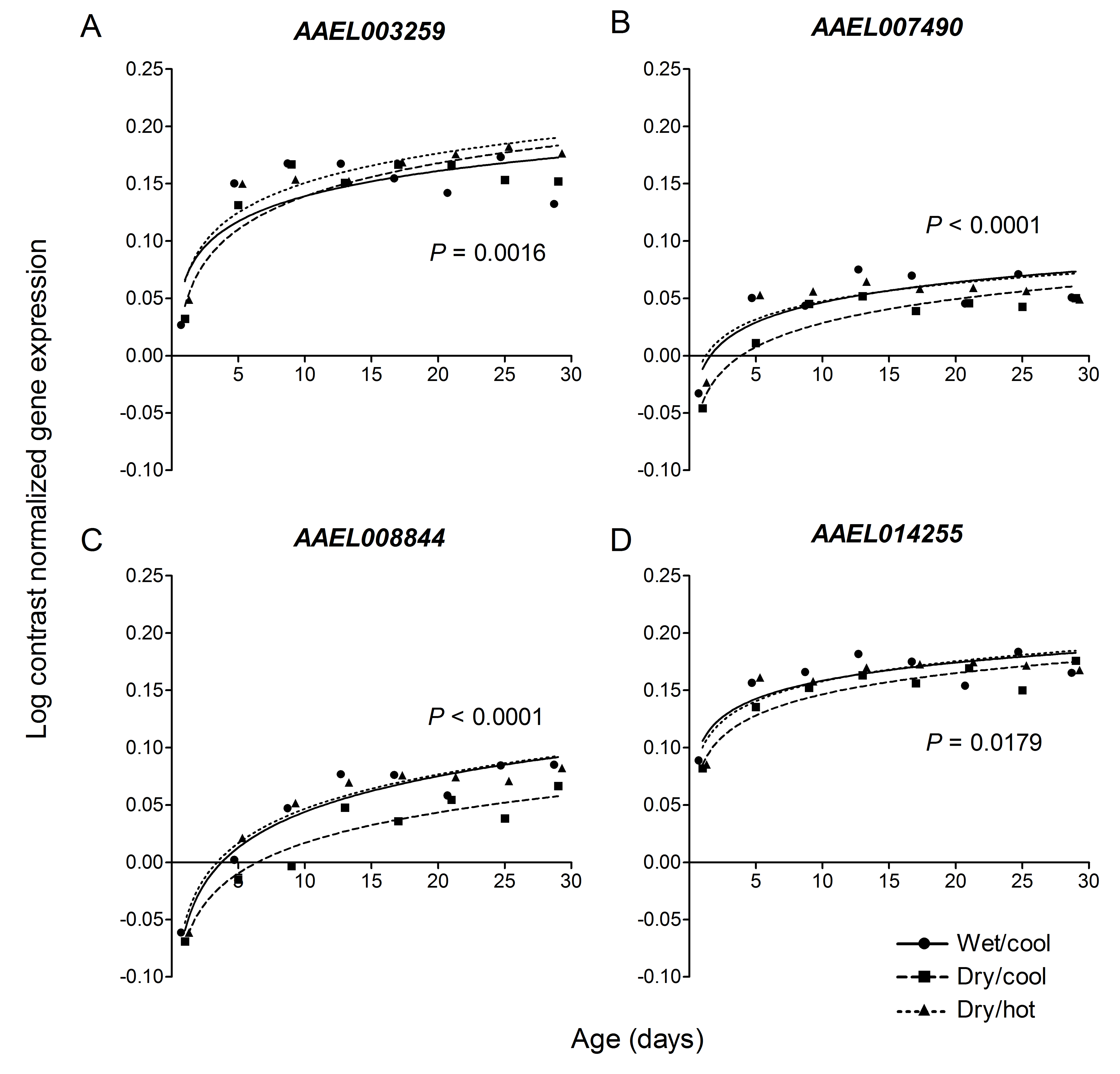

Supplement: Figure S5 — Semi-log curves fitted to the transcriptional profiles of the Tri Nguyen sentinel cage mosquitoes over three seasons. A. AAEL003259 (Pupal cuticle protein 78E). B. AAEL007490 (conserved hypothetical protein). C. AAEL008844 (Calcium binding protein, putative). D. AAEL014255 (Aquaporin, putative). P values indicate the significance of the difference between the regression statistics for individual semi-log models fitted to data for each season (lines) compared to a single model fitted to all data. (TIF) [file pntd.0002669.s005.tif]

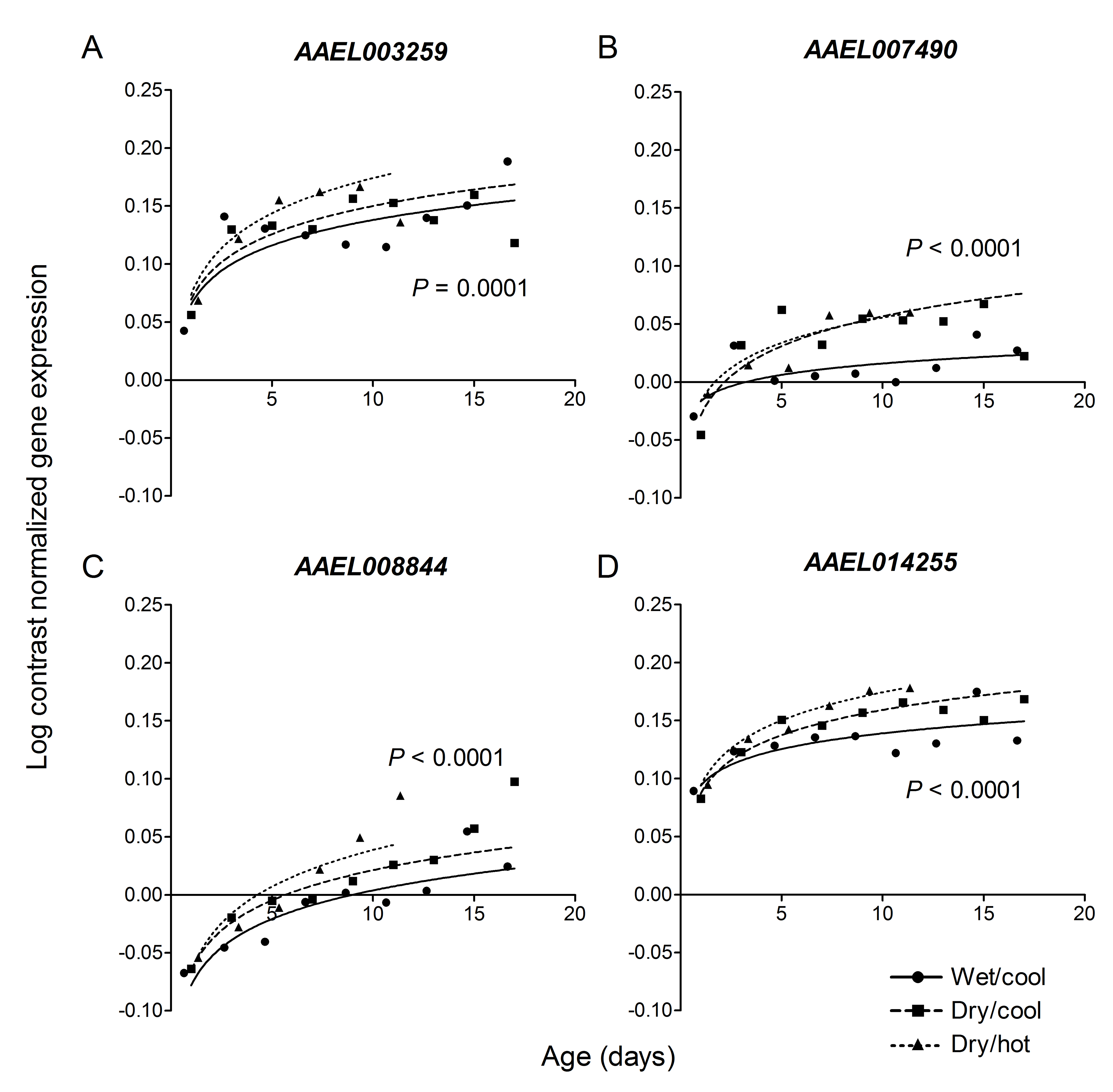

Supplement: Figure S6 — Semi-log curves fitted to the transcriptional profiles of ageing biomarker gene from semi-field small cage mosquitoes over three seasons. A. AAEL003259 (Pupal cuticle protein 78E). B. AAEL007490 (conserved hypothetical protein). C. AAEL008844 (Calcium binding protein, putative). D. AAEL014255 (Aquaporin, putative). P values indicate the significance of the difference between the regression statistics for individual semi-log models fitted to data for each season (lines) compared to a single model fitted to all data. (TIF) [file pntd.0002669.s006.tif]

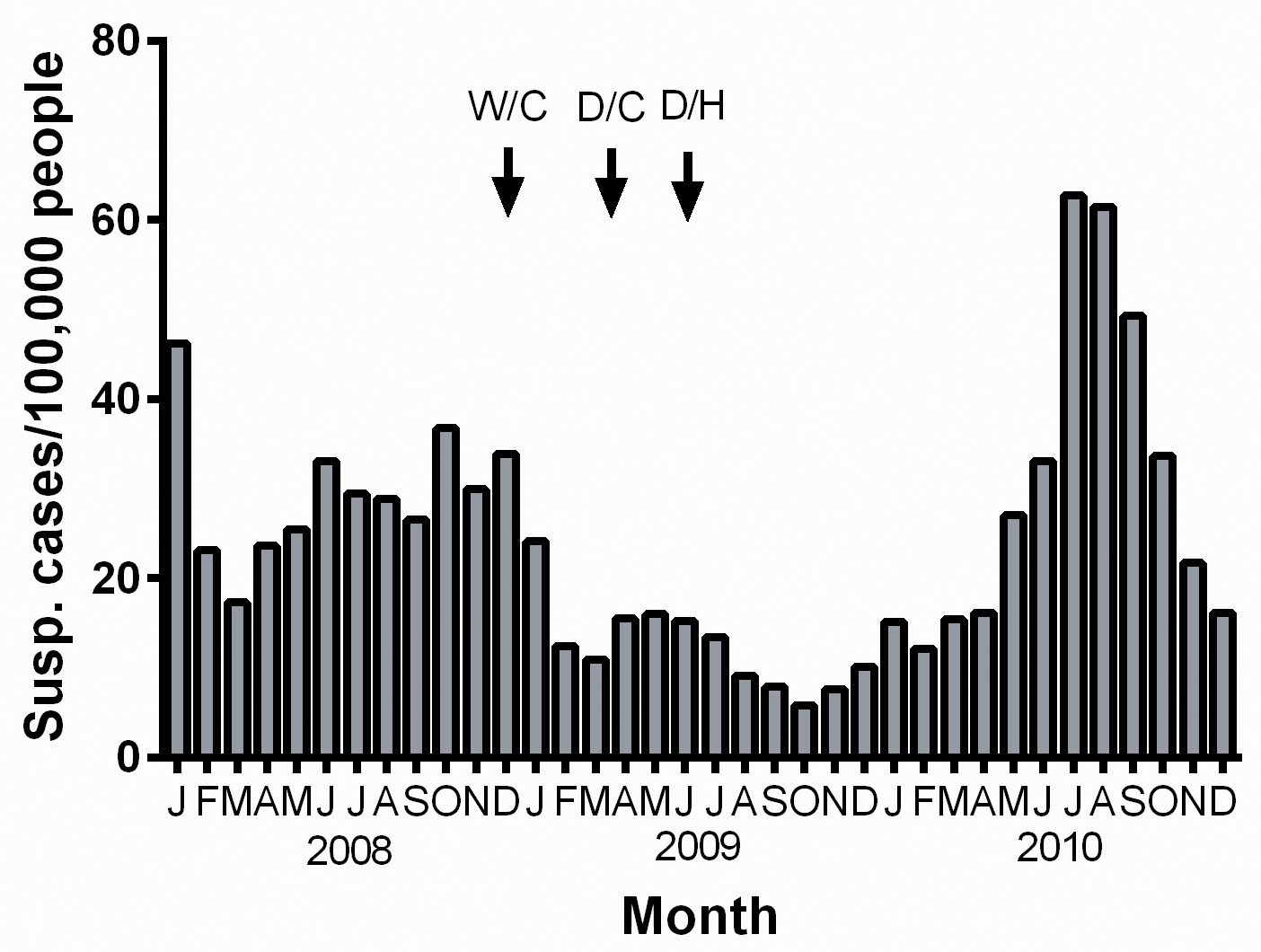

Supplement: Figure S8 — Notifications of suspected dengue cases in Nha Trang, Central Vietnam. Arrows indicate the timing of experiments. W/C; wet/cool season (September-December), D/C; dry/cool season (January-April), D/H; dry/hot season (May-August). (TIF) [file pntd.0002669.s008.tif]
